# Differential Impact of Aging on Cardiovascular Risk in Women Military Service Members

**DOI:** 10.1161/JAHA.120.015087

**Published:** 2020-06-09

**Authors:** Xiaofei Chen, Bala Ramanan, Shirling Tsai, Haekyung Jeon‐Slaughter

**Affiliations:** ^1^ Veterans Affairs North Texas Health Care System Dallas TX; ^2^ Southern Methodist University Dallas TX; ^3^ Department of Surgery University of Texas Southwestern Medical Center Dallas TX; ^4^ Department of Internal Medicine University of Texas Southwestern Medical Center Dallas TX

**Keywords:** cardiovascular risk, predictive model, Veterans Affairs, women, women service members, women veterans, Risk Factors, Women, Cardiovascular Disease, Aging, Race and Ethnicity

## Abstract

**Background:**

Atherosclerotic cardiovascular disease (ASCVD) is the third leading cause of death in women service members and veterans. This study assessed 10‐year ASCVD risk in women service members and veterans using their own electronic health record data extracted from Veterans Affairs (VA) national Corporate Data Warehouse database.

**Methods and Results:**

We retrospectively followed 69 574 VA women, aged 30 to 79 years, from 2007 to 2017. Of these, 52% were whites (n=36 172), 42% were blacks (n=29 232), and 6% were Hispanics (n=4171). Risk factors and ASCVD events (nonfatal myocardial infarction, nonfatal stroke, and cardiac deaths) were identified using diagnostic and procedural codes from electronic health records. Then, within the same construct of the current American College of Cardiology/American Heart Association 10‐year ASCVD risk assessment models for women, coefficients for risks factors were recalculated using the VA national electronic health record data, stratified by race (hereafter, VA women model). Our study found a curvilinear association of aging with increased risk of 10‐year ASCVD event in VA women starting at ages as young as 30 years across all race groups. The VA women model performance in predicting ASCVD events at 10 years was mixed‐moderate in discrimination (C statistics, 0.61–0.64) but good in accuracy, as demonstrated by calibration plots approximating a 45° line.

**Conclusions:**

The study finding, a curvilinear association of aging with increased ASCVD risk in VA women across all races, demonstrates the need for cardiovascular risk screening of younger VA women, aged <45 years.

**Nonstandard Abbreviations and Acronyms**
ACC/AHA: American College of Cardiology/American Heart Association
ASCVD: atherosclerotic cardiovascular disease
CVD: cardiovascular disease
EHR: electronic health record
SBP: systolic blood pressure
VA: Veterans Affairs

**Clinical Perspective**

**What Is New?**

- Our study found that aging was curvilinearly associated with increased 10‐year cardiovascular disease risk in women military service members starting at ages as young as 30 years.

**What Are the Clinical Implications?**

- The study finding may suggest lowering the current recommended age of cardiovascular disease risk screening for women from 45 years to <40 years.

Atherosclerotic cardiovascular disease (ASCVD) is the third leading cause of death in women veterans,^1^ and as such, accurate assessment of ASCVD risk is important not just for prevention and diagnosis,^2^ but also for preoperative workup and operative risk assessment.

Women military service members and veterans have significantly higher number of cardiovascular risk factors and a poorer health status compared with their civilian counterparts.^3^, ^4^ The previous studies^5^, ^6^ reported that women service members had almost twice higher burden of traditional cardiovascular disease (CVD) risk factors, such as hypertension, at younger ages (<40 years) than their civilian counterparts. Currently, women enlists are significantly younger than male enlists in the military. In addition, current and future women service members are more likely to be deployed for combat and to experience multiple deployments than women veterans from the Vietnam and Korean War era. The impact of combat exposures in earlier life can lead to poorer health and ultimately decreased longevity.^7^ Thus, military services in earlier life may alter aging trajectory of ASCVD risk.^8^

This study capitalized on a large, representative Veterans Affairs (VA) national electronic health record (EHR) database and included younger women service members, aged 30 to 40 years, who were previously excluded in the development of the current American College of Cardiology/American Heart Association (ACC/AHA) ASCVD risk assessment model. According to the current ACC/AHA model, women aged <45 years are at a low risk for CVD events.^9^ Consistent with this, the current VA/Department of Defense guideline recommends screening women for CVD risk starting at the age of 45 years, in contrast with men, at the age of 30 years.^10^ Specific to VA women, the traumatic stress associated with military services earlier in life may alter the age trajectory of ASCVD risk later in life.^11^ Therefore, the current ACC/AHA ASCVD risk estimates derived from the general population data^9^ may not be applicable to VA women, who differ in prevalence of traditional and nontraditional CVD risk factors.^4^ To examine this, the study reestimated ASCVD risk for VA women using the VA national EHR data, within the same construct of the current ACC/AHA models.

## Methods

We retrospectively followed 76 559 VA women, non‐Hispanic white, non‐Hispanic black, and Hispanic VA women (women active service members and veterans who received care at VA Health Care system), aged 30 to 79 years, from January 1, 2007, to December 31, 2017. The study selected 76 559 VA women with complete blood pressure data from baseline visit records. Of these VA women, 6985 were excluded from the data analysis because of missing data on cholesterol measures (no lipid panel tests), yielding the final sample size of 69 574. Cholesterol measures dated within the 6‐month period of the baseline visit were selected as baseline cholesterol measures. All variables of interest in the current study were extracted from VA national EHR data, located in VA national Corporate Data Warehouse. The Corporate Data Warehouse data contain health records of all patients treated at nationwide VA Health Care System. These rich and comprehensive VA national EHR data are the best available to study VA population.

Data extraction, preparation, and analyses were performed in the domain of the VA Informatics and Computing Infrastructure. Death event and cause of death data were obtained from the VA Informatics and Computing Infrastructure Vital Status File, which compiles data from the Beneficiary Identification Records Locator Subsystem, death file, and the VA Medicare Vital Status File, and the National Death Index for veterans, which is a part of the VA Suicide Data Repository.

Structural Query Language Server Management Studio (Version 2017; Microsoft Corp, Redmond, WA) was used for data extraction, and statistical and graphical analyses were conducted using SAS Enterprise (Version 7.1; SAS Institute, Cary, NC) and R (Version 3.5.3; cran.r‐project.org), respectively.

Because of the sensitive nature of the VA data collected for this study, requests to access the data set are limited to qualified VA affiliated researchers trained in human subject confidentiality. Protocols may be sent to VA North Texas Health Care System Institutional Review Board at NTXIRBAdmin@va.gov, and Structural Query Language, SAS, and R programming codes that support the findings of this study are available from the corresponding author on reasonable request. The study was approved by the VA North Texas Health Care System Institutional Review Board committee, and no informed consent was required.

CVD risk factors were constructed closely following Sussman and colleagues (2017, Data S1),^12^ and ASCVD event (nonfatal myocardial infarction, nonfatal stroke, and cardiac death) variables were created using *International Classification of Diseases, Ninth Revision* (*ICD‐9*), and *International Classification of Diseases, Tenth Revision* (*ICD‐10*), diagnostic and procedural codes from VA national EHR data and the National Death Index data. In addition, the study checked VA EHR record data accuracy of myocardial infarction and stroke events by searching for words such as “MI,” “myocardial infarction,” and “stroke,” embedded in health providers’ narratives and notes of VA women who experienced such events during the study period.

Then, within the same construct of the ACC/AHA ASCVD risk model,^9^ coefficients of risk factors were recalculated using VA women EHR data, stratified by race (hereafter, VA women model).

Following the same structure of the ACC/AHA model described in Goff et al,^9^ the VA women model included age (natural log transform [Ln] age) and its quadratic form for only white women. The model also included Ln of systolic blood pressure (SBP), and its interaction with antihypertensive treatment for both white and black women, but included a triple interaction term of Ln SBP, antihypertensive treatment, and Ln age for black women only. Total cholesterol (Ln) was included in both race models, but its interaction with Ln age was included in white women only. The current smoking status was included in both race models, but its interaction with Ln age in white women model only. Both race models included Ln of high‐density lipoprotein (HDL), its interaction with Ln age, and presence of diabetes mellitus.

In the ACC/AHA model, separate coefficients were derived for white women and black women; however, no Hispanic women were included. In the current study, new coefficients were calculated for Hispanic women and fitted to both the white and black ACC/AHA models to estimate 10‐year ASCVD risk among Hispanic VA women.

We defined the study assessment points of time as 6‐month visit intervals. If there were multiple visits within 6 months for continuous variables, such as SBP and cholesterol, we averaged multiple values for the variables, and selected a maximum value (1=presence versus 0=no presence) for categorical variables, such as presence of diabetes mellitus, current smoking status, and antihypertensive medication. The first 6 months, January 1 to June 30, 2007, was set to be a baseline visit. However, if there were no visits within 6 months of the June 30, 2007, the first following available visit was set as a baseline visit. We conducted multiple analyses to examine how sensitive results were with different algorithms and methods of missing imputation using Akaike Information Criteria, log likelihood, and residual plots (Table S1).

Harrell's C statistic^13^, ^14^ was used to test a model discrimination of ASCVD events, and calibration plots were used to assess prediction accuracy of VA women model. Proportional hazard assumptions for Cox models were tested for all risk factors using Martingale and Schoenfeld residual plots.

The χ^2^ and t‐statistics were used to examine racial differences in baseline traditional CVD risk factors for categorical and continuous variables, respectively.

Ten‐year ASCVD risk for Hispanic VA women was assessed separately following both white and black VA women models, because the ACC/AHA ASCVD risk models did not include a model specific for Hispanic women.

Relative hazard, known as hazard ratio (HR), of a risk factor was calculated by a simple exponentiation of estimated coefficient, when there was no Ln age interaction term. When Ln age interaction term with a risk factor was included in the model, HR was calculated as a linear combination of both coefficients of the risk factor itself and its interaction term with Ln age, while holding age constant at a mean value. HR >1 is interpreted as increased ASCVD risk, whereas HR <1 is interpreted as decreased risk. The 95% CIs of HR were reported for statistical significance.

## Results

Of the study cohort, 52% were white women, 42% were black women, and 6% were Hispanic women (Table 1). The average age was 46, 44, and 43 years among the white, black, and Hispanic VA women, respectively, and 16% were aged <40 years. Table 1 describes the distribution of baseline CVD risk factors included in the VA women model. SBP, prevalence of diabetes mellitus, and HDL level among black women were significantly higher than in the white and Hispanic women (*P*<0.01; Table 1).

**Table 1:** Baseline Risk Factors, Stratified by Race and Ethnic Group (Total n=69 574)

Variable	Whites (n=36 172 [52%])	Blacks (n=29 231 [42%])	Hispanics (n=4171 [6%])
Age, mean±SD, y	45.86±8.73	44.23±7.82	43.12±8.39
SBP, mean±SD, mm Hg	123.79±14.81	127.09±15.77	122.16±14.55
Diabetes mellitus, n (%)	8405 (23.24)	9569 (32.74)	1056 (25.32)
Current smoking, n (%)	10 864 (30.30)	5111 (17.48)	994 (23.83)
Total cholesterol, mean±SD, mg/dL	200.03±40.77	192.43±38.94	195.57±38.31
HDL, mean±SD, mg/dL	53.82±16.74	56.91±17.38	53.83±15.65

HDL indicates high‐density lipoprotein; and SBP, systolic blood pressure.

There were total of 2176 all‐cause death events (3.1%) among the entire study cohort (white, n=1321 [1.9%]; black, n=781 [1.1%]; Hispanic, n=74 [0.1%]). Table 2 depicted ASCVD events stratified by race and showed that myocardial infarction was the most common ASCVD event, followed by stroke and cardiac death. The rate of stroke was significantly higher in black women (2.0%) than white women (1.5%, *P*<0.01; Table 2).

**Table 2:** Number of CVD Events by Race and Ethnicity

CVD Events^a^	White, n (%)	Black, n (%)	Hispanic, n (%)
Nonfatal myocardial infarction^b^	1515 (4.2)	1148 (3.9)	148 (3.6)
Nonfatal stroke^c^	538 (1.5)	592 (2.0)	61 (1.5)
Cardiac death^d^	235 (0.6)	151 (0.5)	15 (0.4)

CVD indicates cardiovascular disease.

a The same patient can experience multiple CVD events.

b White women>black women>Hispanic women, *P*=0.06.

c Black women>white, Hispanic women, *P*<0.01.

d White women>black women>Hispanic women, *P*=0.01.

**Figure 1:**
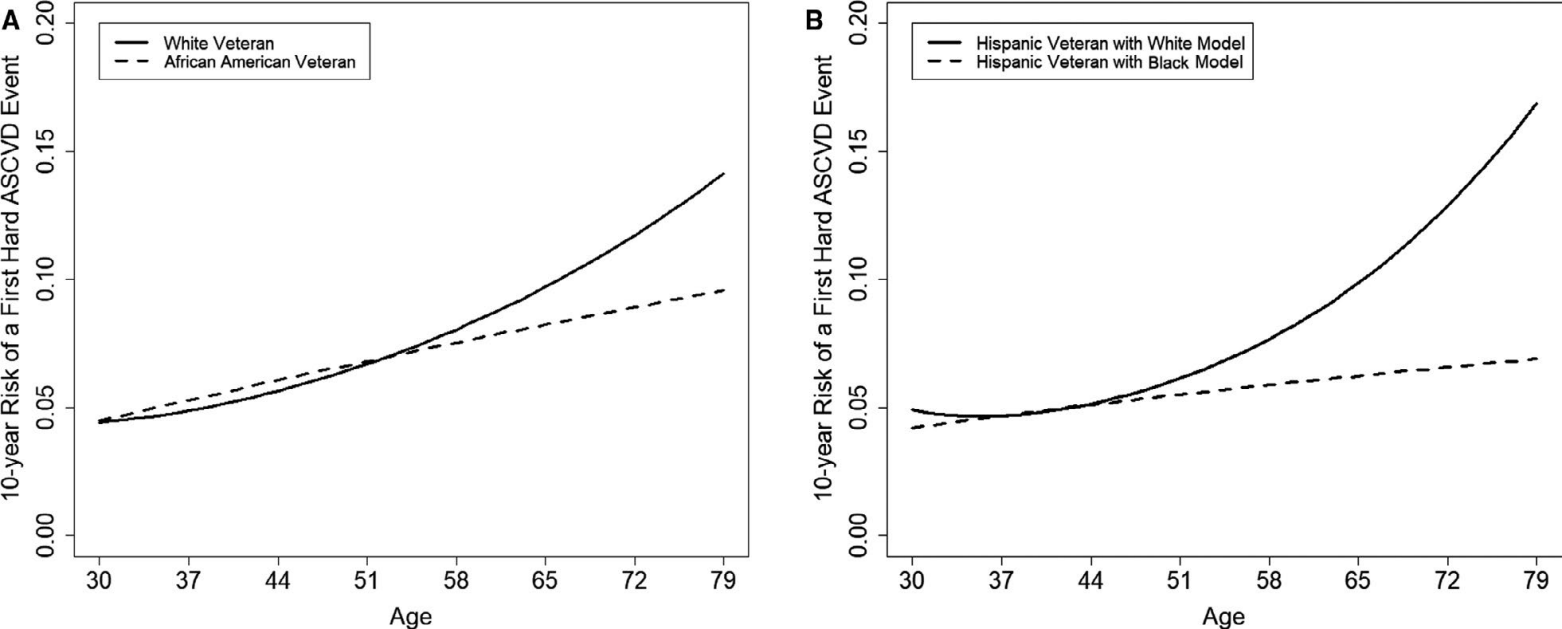
Aging effect on increased 10‐year atherosclerotic cardiovascular disease risk (ASCVD), stratified by race between civilian women and women military service members. **A**, White and black women. **B**, Hispanic Veterans Affairs women. American College of Cardiology/American Heart Association white and black women model structures were followed. *Solid lines represent white women ASCVD risk assessment model; dashed lines represent the black women model.

The estimated 10‐year ASCVD risk for VA women increased curvilinearly with older age, starting at the age of 30 years in both white and black VA women (Figure 1A). Figure 1B showed a similar curvilinear association of increased ASCVD risk with aging among Hispanic women in each model, white and black.

C‐statistics for the VA women models were 0.64 for the whites, 0.63 for the blacks, and 0.61 for the Hispanics. The VA women model explained 82% of the variance of predicted CVD events among VA white women. Contrary to the white women, predictive accuracy of the models for VA black and Hispanic women diminished with inclusion of traditional CVD risk factors, yielding negative explained variances (blacks, −8%; and Hispanics, <−120% in each race model).

The baseline survival probabilities at 10 years were 0.941, 0.939, and 0.949 for the white, black, and Hispanic VA women, respectively. Estimated 10‐year ASCVD risks were 5.1% and 5.2% for the white and black women, respectively, at the age of 50 years, total cholesterol was 203 mg/dL, HDL was 50 mg/dL, SBP was 120 mm Hg, no diabetes mellitus, and no current smoking (Table S2).

For Hispanic VA women, the current study used both white and black women ACC/AHA models to estimate 10‐year ASCVD risk, and they were 5.1% and 5.2%, respectively, at the age of 50 years, total cholesterol was 203 mg/dL, HDL was 50 mg/dL, SBP was 120 mm Hg, no diabetes mellitus, and no current smoking (Table S2).

Table 3 showed estimated coefficients of CVD risk factors included in the VA women model, stratified by race.

**Table 3:** Estimates of VA Women ASCVD Model by Non‐Hispanic White, Non‐Hispanic Black, and Hispanic Women

Variable	White		Black		Hispanic (White Model)		Hispanic (Black Model)	
	Estimate	SE	Estimate	SE	Estimate	SE	Estimate	SE
Ln age	−8.476	7.397	1.662	6.078	−14.331	24.388	3.070	16.172
Ln age^2^	1.031	0.542	···		2.087	1.775	···	
SBP untreated	0.711	0.193	2.580	7.528	0.447	0.631	−5.396	19.356
Ln SBP untreated×Ln age	···		−0.341	1.191	···		0.938	3.077
SBP treated	0.012	0.011	−1.294	0.406	0.013	0.036	−0.672	1.287
Ln SBP treated×Ln age	···		0.207	0.064	···		0.108	0.203
Diabetes mellitus	0.113	0.051	0.183	0.051	0.203	0.159	0.213	0.159
Current smoking	−1.827	1.602	0.001	0.061	5.773	5.045	0.119	0.157
Current smoking×Ln age	0.295	0.253	···		−0.902	0.806	···	
Ln total cholesterol	0.344	3.563	0.245	0.119	5.203	11.272	0.569	0.356
Ln total cholesterol×Ln age	−0.028	0.560	···		−0.734	1.788	···	
Ln HDL	4.058	2.503	−2.398	2.978	10.443	7.903	10.457	8.121
Ln HDL×Ln age	−0.818	0.395	0.199	0.472	−1.768	1.262	−1.769	1.297
C statistics	0.639		0.630		0.618		0.614	

ASCVD indicates atherosclerotic cardiovascular disease; HDL, high‐density lipoprotein; Ln, natural log transform; SBP, systolic blood pressure; and VA, Veterans Affairs.

Presence of diabetes mellitus increased ASCVD risk for the white and black VA women by 12% and 20%, respectively (whites: HR, 1.12; 95% CI, 1.01–1.24; blacks: HR, 1.20; 95% CI, 1.09–1.33; Table 3). The VA white women's ASCVD risk doubled with 1–mm Hg increase of untreated SBP at mean age (HR, 2.03; 95% CI, 1.40–2.97; Table 3), whereas risk also increased in other race VA women (blacks: HR, 1.57; 95% CI, 1.02–2.41; Hispanic VA women, white and black models: HR, 1.56; 95% CI, 0.45–5.38; and HR, 1.61; 95% CI, 0.43–5.70, respectively; Table 3). With the increase of the total cholesterol level by 1 mg/dL, ASCVD risk evaluated at mean ages increased across all race and ethnic groups (whites: HR, 1.18; 95% CI, 0.94–1.47; blacks: HR, 1.28; 95% CI, 1.01–1.61; Hispanics under white and black models: HR, 1.85; 95% CI, 0.89–3.94; and HR, 1.77; 95% CI, 0.88–3.55, respectively).

As HDL level increased by 1 mg/dL, the 10‐year ASCVD risk decreased in both white (HR, 0.33; 95% CI, 0.29–0.39; Figure S1) and black VA women (HR, 0.32; 95% CI, 0.27–0.37), holding age constant at mean values. Active smoking increased ASCVD risk with older age among VA white women (HR, 1.04; 95% CI, 0.94–1.13; Figure S2), but its effect was close to zero among black VA women (HR, 1.00; 95% CI, 0.89–1.13).

**Figure 2:**
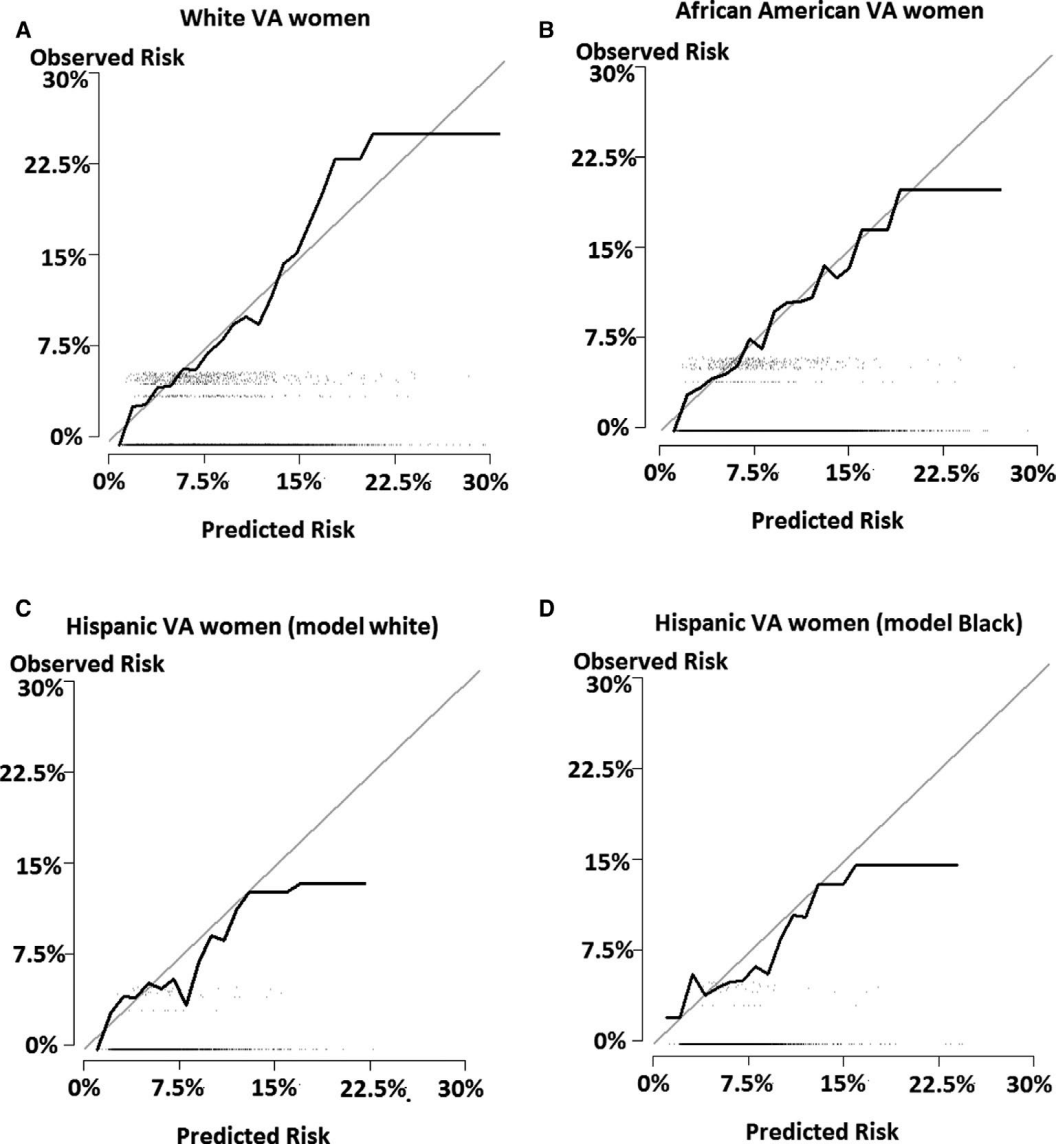
Calibration plots for Veterans Affairs (VA) women atherosclerotic cardiovascular disease (ASCVD) risk model by race and ethnicity. **A**, White VA women. **B**, Black VA women. **C**, Hispanic VA women (white model). D, Hispanic VA women (black model). *A 45° gray line represents a perfect agreement between observed and predicted ASCVD risk probabilities.

Figure 2 showed calibration plots of observed and predicted probabilities of ASCVD events with a 45° line representing a perfect agreement between observed and predicted probabilities. Overall, the VA women model predicted ASCVD events close to the observed probability up to 15% for white women (Figure 2A) and up to 20% for black women (Figure 2B).

For Hispanic women, both race models slightly overpredicted ASCVD events, albeit there was a good agreement between predicted and observed risk probabilities. However, discrepancies between predicted and observed CVD risk probabilities in Hispanic VA women widened at 12% and higher observed probability (Figure 2C and 2D).

## Discussion

**Figure 3:**
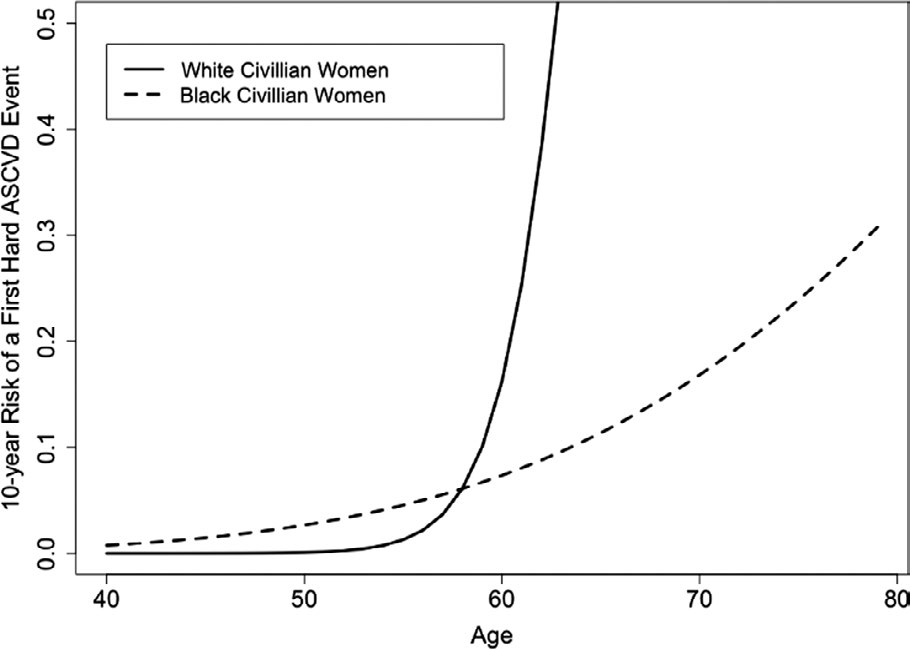
Aging effect on atherosclerotic cardiovascular disease (ASCVD) risk: the current American College of Cardiology/American Heart Association model. *A solid line represents civilian white women ASCVD risk assessment model; a dashed line represents the civilian black women model. ^†^The ASCVD score was originally developed using a pooled cohort data set created from 5 large National Institutes of Health–funded epidemiological cohort data and they are the Framingham Study,^15^ the Framingham Offspring Study,^16^ ARIC (Atherosclerosis Risk in Communities),^17^ CHS (Cardiovascular Health Study),^18^ and CARDIA (Coronary Artery Risk Development in Young Adults).^19^ Numbers of white and black women in this cohort were 11 240 and 2641, respectively. The estimated risk coefficients for natural log transform (Ln) age and Ln age^2^ for white women were −29.799 and 4.884, respectively, whereas estimated Ln age coefficient for black women was 17.114. For details, the data description is given by Goff et al^9^ in Appendix 7, Tables A and B.

Our study found that VA women's 10‐year ASCVD risk increased steadily with older age from the age of 30 years across all race groups, contrary to the current ACC/AHA model's differential aging effect by race (Figure 3).^15^, ^16^, ^17^, ^18^, ^19^ VA women's 10‐year ASCVD risk was estimated higher for women aged <50 years than their civilian peers. The ASCVD risk among VA white women increased curvilinearly with older age, starting from as early as the age of 30 years (Figure 1A), while at a minimum risk until the age of 50 years but escalating after the age of 50 years, J‐shape aging trajectory of ASCVD risk, in civilian counterparts (Figure 3). This finding supports the study's hypothesis, military service in earlier life may alter aging trajectories of ASCVD risk, considering a military service exposure as a natural experiment, whereas other CVD factors are equal between VA and civilian women.

One of the critiques of the current ACC/AHA model is an overestimation of aging effect on ASCVD risk for the population aged >55 years and underestimation of the population aged <40 years. An application of the ACC/AHA model to VA women aged 40 to 79 years also supported these critiques (Table S3 and Figure S3). With inclusion of a substantial number of younger women, and capitalizing on large‐scale, EHR data, the VA women model may have corrected overestimation of ASCVD risk among the older female population and underestimation of the risk among the younger female population.

However, inclusion of a substantial number of VA women aged <40 years may account for a finding of higher 10‐year ASCVD risk among VA women than their civilian peers. The current study cohort had much lower mean ages, 46 and 44 years for white and black VA women, respectively, than civilian women from the pooled cohort data used to develop the original ACC/AHA model (mean age, 54 and 52 years for white and black civilian women, respectively).

The current study reported new 10‐year ASCVD risk assessment for Hispanic VA women following both white and black women ACC/AHA model structures. The sample size of Hispanic VA women data used for estimation in the current study was 4575, which is small but equivalent to the original ACC/AHA black civilian women cohort data. Figure 1A and 1B showed curvilinear aging effects on increase in ASCVD risk among Hispanic VA women following white women model and a linear aging effect following black women model. These were similar with white and black VA women results, except slightly larger effects.

The study findings suggest that the aging effect on ASCVD risk among VA women may be similar across all race VA women, curvilinear effect (Figure 1), rather than aging effect differentiated by race (Figure 3), as suggested in the current ACC/AHA model. The current ACC/AHA women model structures differentiate black women from white women, in particular, with inclusion of interaction terms with Ln age. This may be partly because of the smaller sample size of the development cohort data in certain age groups, such as aged <45 years and >65 years.

Overall, the VA women model found that relative hazards of traditional risk factors were much smaller than those reported in the current ACC/AHA women model. This was likely because of larger‐scale data used to estimate VA women model. With larger‐scale data, possible overestimation of relative hazards is expected to be corrected.

Despite the advantage of the large‐scale data, the EHR data are often criticized on possible misclassifications of *ICD‐9* and *ICD‐10* diagnosis codes. This weakness can be mitigated by validating *ICD* codes against providers’ narrative notes from medical records. This study defined CVD events, such as nonfatal myocardial infarction and nonfatal stroke, from *ICD‐9* and *ICD‐10* diagnosis and procedure codes. The accuracy of nonfatal myocardial infarction event, on the basis of *ICD* codes from VA EHR, has been provided to be good (96.9% concordance) against providers’ notes in the previous studies.^20^, ^21^ Although some studies found *ICD* diagnosis codes for stroke events inaccurate (50%–61% concordance with providers’ notes),^22^, ^23^ the current study found a high accuracy, 92.5% concordance between stroke *ICD‐9* and *ICD‐10* diagnosis and procedural codes and providers’ notes, in our study cohort. Thus, the accuracy of CVD events among VA women on the basis of *ICD* codes from VA EHR data is acceptable.

Performance of the VA women model, measured by explained variance, prediction accuracy (C‐statistics), and a model fit (calibration plots), was mixed. Explained variance of the VA women model was high, >80%, for white VA women, whereas it was poor for both black and Hispanic women (negative explained variances). Despite calibration plots that demonstrated a good fit of the VA women model (Figure 2), the model produced a moderate prediction accuracy under C statistics, 0.61 to 0.64. In other words, the model would correctly classify ASCVD events 61 to 64 times of 100 times. Lower CVD event rates and a high proportion of censored observations may account for negative explained variation of the model; however, negative explained variation does not necessarily indicate a poor model performance of the model, such as moderate C statistics in the current study.^24^ These moderate C statistics for all 3 race and ethnicity models suggest a potential underestimation of 10‐year CVD risk in VA women from omitting important CVD risk factors. The accuracy of the model prediction could be improved by adding nontraditional CVD risk factors, such as major depression,^4^ military service characteristics, such as number of deployments^25^ or length of service, and recalibration of age variable, removal of interaction terms with Ln age from the model, supported by the study finding, a curvilinear aging effect on increased ASCVD risk starting as early as the age of 30 years.

Our study is not the first study that developed CVD risk prediction model for VA women using VA EHR data. VA Cardiac Risk Score is a previous study that developed a CVD risk predictive model for VA women using VA EHR data.^12^ However, the VA Cardiac Risk Score used different model and estimation approaches from the current study. First, the VA Cardiac Risk Score was not stratified by race because of a small sample size of black VA women and developed one model fit for all races with a race covariate, a binary indicator, black versus nonblack VA women. Thus, the model structure of the VA Cardiac Risk Score is different from race‐stratified ACC/AHA women model in the current study. Second, the VA Cardiac Risk Score applied logistic regression model, whereas the ACC/AHA women models used time‐to‐event analysis, Cox proportional model.

The ACC/AHA model used Cox proportional hazard model under the assumption that right censoring is not informative of ASCVD event outcomes (ie, right censoring is independent of ASCVD event outcomes). However, this is untestable hypothesis under the current study. And a right censoring in VA EHR data could imply loss to follow‐up of VA women when women military service members sought treatment elsewhere at non‐VA healthcare settings. If women service members and veterans with multiple CVD risk factors were more likely to seek healthcare services outside of VA health system, then right censoring is informative, and thus, will violate the assumption of independence. In such a case, the standard Cox proportional hazard model estimation will be biased (Binder).^26^ Future studies using inverse propensity score weighted Kaplan‐Meier and g‐estimation methods, proposed by Robins and colleagues, would correct a potential bias in estimation caused by right censoring.^27^

Limitations are noted. The current study estimated cardiac death using cause of death data available from National Death Index 2007 to 2016. Thus, it is possible that the current number of cardiac deaths may have been underreported because of no data availability on cause of death in 2017. However, the number of cardiac deaths reported in this study is compatible with the previous study^12^; thus, a bias in estimation from omitting cardiac death in 2017 is expected to be minimum.

The current study is limited to VA women with complete data on vital signs, SBP, and total cholesterol and HDL at baseline visits, which may result in a sampling bias. Despite a potential sampling bias, this ensures that the study cohort VA women were patients who actually received treatment at VA healthcare system by confirming visit records with vital sign data and blood pressure. Analyzing the cohort with complete cholesterol data is essential to adhere to the ACC/AHA model structure and estimation methods in predicting 10‐year CVD event risk for VA women using VA EHR data.

VA women aged <30 and >80 years were excluded from the current study cohort.

In conclusion, this model demonstrates a new relationship between age and CVD risk in women veterans. The findings emphasize the need to reevaluate the current VA/Department of Defense CVD screening age guideline for women. The current VA/Department of Defense guideline recommends a CVD screening for women at the age of 45 years, whereas screening at the age of 30 years is recommended for men.^10^ The study finding may suggest lowering the current recommended age of CVD risk screening for women from 45 to <40 years.^10^, ^28^ A future study is warranted to develop a single, consistent ASCVD risk assessment model that fits across all race and ethnic women.

## Sources of Funding

The study is funded by the Department of Defense Peer‐Reviewed Medical Research Discovery Award (W18XWH1810159) and Jeon‐Slaughter, PhD, is the study principal investigator. The views, opinions, and/or findings contained in this article are those of the authors and should not be construed as an official Department of the Army position, policy, or decision unless so designated by other documentation.

## Disclosures

None.

## Supporting information


**Data S1**
**Tables S1–S3**
**Figures S1–S3**
**Reference 12**
