# Supplementary material for: Differential Impact of Aging on Cardiovascular Risk in Women Military Service Members
Source: J Am Heart Assoc. 2020 Jun 9;9(12):e015087. doi: 10.1161/JAHA.120.015087 (PMC7429070; doi:10.1161/JAHA.120.015087)
Supplement: Supplementary file 1 — Data S1 Tables S1–S3 Figures S1–S3 Reference 12 [file JAH3-9-e015087-s001.pdf]

Supplemental Material

Data S1.

Study cohort and variable construction

1.A. Section criteria to construct Veterans Affairs (VA) women cohort

The current study employed a strategy selecting the study cohort, Veterans Affairs (VA) women, for those with complete data on vital signs and laboratory results available at baseline visits. By taking an advantage of large electronic health records from VA system, VA women patients have multiple visit records with corresponding dates separately for ambulatory care, inpatient care, laboratory test orders and results, medication dispense, and problem lists with the known existing chronic conditions with onset or earliest record dates.

Due to inherent characteristics of EHR records, there exist multiple visit records with different dates of vital signs and laboratory results (such as lipid panel results) and medication records per patient and some of these records have incomplete data. Our study strategy to deal with this issue of EHR records was not to impute incomplete data on vital signs such as blood pressure and cholesterol values when they are not available during the study period. Under this strategy, we defined VA women as those who actually treated at VA health care system by confirming visit records with vital sign data, Blood pressure data, recorded and entered during the visit. The VA standard treatment procedure guideline for VA outpatient and inpatient visits is to measure, record, and enter blood pressure values into medical records. Thus, if blood pressure data are missing from the visit record we can assume the patient may not have treated at VA health care system.

For missing data on cholesterol data, we restricted our study cohort to those patients with complete cholesterol data since the ACC/AHA model structure is following the cholesterol model and having complete cholesterol is essential for ACC/AHA model. When either total cholesterol or high density lipoprotein (HDL) cholesterol was missing but other lipid panel test results such as Low Density Lipoprotein (LDL)-cholesterol and triglyceride were available from a lipid panel test and units, mg/dL and mmol/L, we then calculated them by applying a formula, total cholesterol= HDL + LDL + 1/5 triglyceride.

1.B. Cardiovascular disease (CVD) risk factors, diabetes, current smoking and antihypertensive medication treatment

With the VA women cohort with complete systolic blood pressure (SBP) and cholesterol data for baseline visits, we constructed CVD risk factors with a binary value, such as diabetes, current smoking, and antihypertensive medication status using ICD 9 and 10 codes, laboratory results, health factors and pharmacy records. When data were available and meeting criteria, these CVD risk factors were recoded as "Yes." The criteria to meet diabetes condition were both diagnosis ICD codes (see Sussman et al 2017[12] Supplemental materials) and HbA1C cut off value ≥ 6.5% (48mmol/mol) and we used within 6 month medication dispense date of antihypertensive medication.[12] When unknown or missing data on diabetes condition and medication dispense dates we imputed as "No." This is known as a first order missing imputation and a valid and common method in constructing variables.

Calculating current smoking status variable used a different strategy. It employed using Health Factor type (smoking status type) data available within VA EHR. First we tabulated all possible entry of smoking status and If there were no records in health factors smoking type data,

the VA woman was then recorded as “no current smoking.” For those with records with non NULL entry for smoking status type, we tabulated all unique entries and selected the following contents to define current smoking status (Please see the below for the details). When a selected narrative smoking status was indicated from visit dates within 6 months of the baseline visits, the study recorded her as “Current smoking” otherwise, the study recorded her as “No current smoking.”

Narratives selected to define “Current smoking” are:

"ADVISED TO REMOVE TOBACCO PROD HOME/WORK",
"ADVISED TO SET A QUIT DATE WHEN READY",
"BH WARD TOBACCO CESSATION GROUP",
"BRIEF INTERVENTION PHYSICIAN TOBACCO",
"CESSATION MEDICATIONS",
"CIGARETTE USER",
"CURRENT SMOKELESS TOBACCO USER",
"CURRENT SMOKER",
"CURRENT TOBACCO USE",
"CURRENT TOBACCO USER",
"CURRENT TOBACCO USER (VERIFIED)",
"CURRENT TOBACCO USER ON SCREEN",
"CURRENTLY ENROLLED IN SMOKING CESSATION",
"Current tobacco user",
"DISCUSSED REASONS/BENEFITS OF QUITTING",
"HF V9 CURRENT SMOKER",
"I-CURRENT SMOKER",
"INPATIENT CURRENT TOBACCO USER",
"INPT INFORMED OF TOBA CCO RISK",
"INPT QUIT SMOKING COUNSELING",
"INPT QUIT SMOKING STRATEGIES",
"INPT SMOKES CIGARETTES >=5 DAILY",
"INPT TOBACCO MEDS OFFERED-ACCEPTED",
"INPT TOBACCO SCREENING",
"KC-TOBACCO CESSATION CLINIC REQUESTED",
"LOM Inpt Current Smoker",
"MED CURRENT SMOKER",
"NICOTINE LOZENGE TAPERED 4MG",
"NICOTINE PATCH 21MG-7MG",
"NICOTINE PATCHES PRESCRIBED BY PCP",
"NO LONGER DESIRES MEDS FOR TOB CESS",
"NSG CURRENT SMOKER PAST 30 DAYS",
"NSG TOBACCO COUNSELING RECEIVED",
"NURSING: TOBACCO MEDS OFFERED-ACCEPTED",
"OFFERED NICOTINE DEPENDENCE CLINIC",
"OFFERED NICOTINE REPLACEMENT BUT REFUSED",
"OFFERED STOP TOBACCO CLINIC REFERRAL",

"PATIENT DECLINED (SMOKING CESSATION ED)",
"PHI TOBACCO CURRENT USER",
"POSITIVE TOBACCO SCREEN",
"PRESCRIBED TOBACCO CESSATION MEDICATION",
"PRIMARY CARE TOBACCO CESSATION PROGRAM",
"PROVIDER CURRENT SMOKER",
"PROVIDER ORDER TOBACCO MEDS",
"PROVIDER TOBACCO COUNSELLING DONE",
"PT DECLINES REF TO TOBACCO CESS PRGM",
"PT DECLINES TOBACCO CESSATION MEDS",
"PT NOT INTERESTED IN QUITTING TOBACCO",
"PT READY TO QUIT TOBACCO USE",
"PT REFUSES TOBACCO CESSATION PROGRAM",
"PT REQ NICOTINE PATCH",
"PT THINKING ABOUT QUITTING TOBACCO USE",
"Patient requests Nicotine Gum",
"QUIT SMOKING MEDICATION REFERRAL",
"QUIT TOBACCO <12 MONTHS AGO",
"QUIT TOBACCO IN LAST 12 MOS.(VERIFIED)",
"QUIT TOBACCO IN THE LAST 12 MONTHS",
"QUIT TOBACCO USE > 7 YEARS AGO",
"QUIT TOBACCO USE IN LAST YEAR",
"QUIT TOBACCO USE IN PAST YEAR",
"QUIT TOBACCO USE WITHIN LAST YEAR",
"REFUSED SMOKING CESSATION",
"REFUSED SMOKING CESSATION REFERRAL",
"REFUSES SMOKING CESSATION",
"SMOKELESS TOBACCO USER",
"SMOKER - OFFERRED MEDS (PROVIDER)",
"SMOKING (Q4L) ENROLLED",
"SMOKING CESSATION CLINIC NOTE DONE",
"SMOKING CESSATION MEDICATION",
"SMOKING CESSATION REFERRAL",
"SMOKING MEDICATION INTEREST",
"SUSPEND TOBACCO COUNSELING",
"TOB COUNSEL BY PROVIDER",
"TOB INFO ON NON-VA STOP SMOKING CLINIC",
"TOB INTERESTED IN CESS MEDS",
"TOB MEDS DECLINED",
"TOB MEDS PRESCRIBED",
"TOB NON-TRADITIONAL PRESCRIBER",
"TOB OTHER INTERVENTIONS",
"TOB Offered Pt Meds (Provider) FY09",
"TOB QUIT LATER (SCREEN)",
"TOB REFER TO TELEQUIT",
"TOB Rx arranged",

"TOB Rx non-VA meds",
"TOB Rx not now",
"TOB Rx prescribed",
"TOB SC non-VA",
"TOB SC will call to enroll i/SC",
"TOB USE BUPROPION ORDERED",
"TOB USE COUNS APPT",
"TOB USE COUNS PHONE",
"TOB USE NICOTINE REPLACEMENT",
"TOB contemplating quitting",
"TOB declined TOBACCO CESSATION",
"TOB resists SC",
"TOB strategies & plan",
"TOB-DECLINES SMOKING CESSATION REFERRAL",
"TOBACCO ADM RN CONSULT-INPT-PHARM",
"TOBACCO ADVISED PATIENT TO QUIT",
"TOBACCO ALTERNATIVE - NICOTINE PATCH/GUM",
"TOBACCO ASSISTED WITH CESSATION",
"TOBACCO BARRIERS TO QUITTING REVIEWED",
"TOBACCO CESSATION ALREADY ENROLLED",
"TOBACCO CESSATION BENEFITS REVIEWED",
"TOBACCO CESSATION CLINIC ADDRESSED",
"TOBACCO CESSATION CLINIC NO",
"TOBACCO CESSATION CLINIC OFFERED",
"TOBACCO CESSATION CONSULT ORDERED",
"TOBACCO CESSATION COUNSELING NURSE",
"TOBACCO CESSATION GROUP BCVAMC",
"TOBACCO CESSATION MEDICATION ACCEPTED",
"TOBACCO CESSATION MEDICATION DECLINED",
"TOBACCO CESSATION MEDICATION NEED RX",
"TOBACCO CESSATION MEDICATION OFFERED",
"TOBACCO CESSATION MEDICATION ORDERED",
"TOBACCO CESSATION MEDICATION PREVIOUS RX",
"TOBACCO CESSATION MEDICATION REFUSED",
"TOBACCO CESSATION MEDICATIONS",
"TOBACCO CESSATION MEDS - ALREADY ON",
"TOBACCO CESSATION MEDS ADDRESSED",
"TOBACCO CESSATION MEDS NO",
"TOBACCO CESSATION MEDS OFFERED",
"TOBACCO CESSATION MEDS ORDERED",
"TOBACCO CESSATION MEDS REFUSED",
"TOBACCO CESSATION OFFERED",
"TOBACCO CESSATION OFFERED/DECLINED",
"TOBACCO CESSATION PROGRAM ENROLLED",
"TOBACCO CESSATION PROGRAM REFERRAL ORDER",
"TOBACCO CESSATION REFERRAL DECLINED",

"TOBACCO CESSATION REFERRAL OFFERED",
"TOBACCO CESSATION REFERRAL REFUSED",
"TOBACCO CESSATION STRATEGIES DISCUSSED",
"TOBACCO CESSATION THERAPY ONGOING",
"TOBACCO CHANTIX NO EXCLUSIONS",
"TOBACCO CHANTIX NO MH DX",
"TOBACCO CONSULT-OUPT-DISCHARGE",
"TOBACCO CONTROLLING MEDS OFFERED",
"TOBACCO COUNSELING & OFFERED REFERRAL",
"TOBACCO COUNSELING 1",
"TOBACCO COUNSELING CONTRAINDICATED",
"TOBACCO COUNSELING DISCHARGE INPT DONE",
"TOBACCO COUNSELING DONE",
"TOBACCO COUNSELING DONE BY NURSE",
"TOBACCO COUNSELING INPATIENT",
"TOBACCO COUNSELING OFFERED",
"TOBACCO COUNSELING REFUSED",
"TOBACCO COUNSELING, REFUSED TO QUIT",
"TOBACCO CURRENT USER",
"TOBACCO DECLINED MEDS",
"TOBACCO DISCHARGE POS USE",
"TOBACCO INPATIENT COUNSELING GIVEN",
"TOBACCO INPATIENT MEDS REFUSED",
"TOBACCO INPATIENT REFERRAL REFUSED",
"TOBACCO INPT CONSULT-PHARM DECLINED",
"TOBACCO INQUIRY POSTITVE",
"TOBACCO INTERVENTION REFUSED AT D/C",
"TOBACCO MEDICATION ORDERED",
"TOBACCO MEDICATION REFERRAL DECLINED",
"TOBACCO MEDICATION REFERRAL YES",
"TOBACCO MEDICATION REFUSAL",
"TOBACCO MEDICATIONS OFFERED",
"TOBACCO MEDS ADDRESSED",
"TOBACCO MEDS NON PROVIDER",
"TOBACCO MEDS NOT NECESSARY (<5CIG/DAY)",
"TOBACCO MEDS OFFERED",
"TOBACCO MEDS OFFERED BUT DECLINED",
"TOBACCO MEDS OFFERED/DECLINED",
"TOBACCO MEDS REFUSED",
"TOBACCO NO REFERRAL",
"TOBACCO NON USE LESS THAN 12 MONTHS",
"TOBACCO OFFER MEDS-USING NON VA PRODUCT",
"TOBACCO OFFERED CESSATION REFERRAL",
"TOBACCO OFFERED MEDS NON-PROVIDER",
"TOBACCO OFFERED MEDS OTHER",
"TOBACCO OFFERED MEDS PT REFUS (NON-PROV)",

"TOBACCO OFFERED PT MEDS (CLINICIAN)",
"TOBACCO OFFERED PT MEDS (MD/NP/PA)",
"TOBACCO OFFERED PT MEDS (PROVIDER)",
"TOBACCO OFFERED PT MEDS (PROVIDER)1",
"TOBACCO OFFERED REFERRAL (PROVIDER)",
"TOBACCO OFFERED STOP SMOKING CLINIC",
"TOBACCO OFFERED STOP SMOKING CLINIC1",
"TOBACCO OFFERED STOP SMOKING MEDS",
"TOBACCO OFFERRED PT MEDS (PROVIDER)",
"TOBACCO OFFERRED STOP SMOKING CLINIC",
"TOBACCO OUTPATIENT MEDS ORDERED",
"TOBACCO PACK YEARS <30",
"TOBACCO PACK YEARS >29",
"TOBACCO PAST ATTEMPTS TO QUIT REVIEWED",
"TOBACCO PAST MONTH-INPT-ADM",
"TOBACCO PAST MONTH-INPT-DISCHARGE",
"TOBACCO PATIENT ACCEPTS CLINIC",
"TOBACCO PATIENT ACCEPTS MEDS",
"TOBACCO PATIENT ACCPEPTS MEDS RN",
"TOBACCO PATIENT DID NOT RECEIVE MEDS",
"TOBACCO PATIENT REFUSE MEDS",
"TOBACCO PATIENT REFUSED CLINIC",
"TOBACCO PATIENT REFUSES TO QUIT",
"TOBACCO PCP COUNSELLING NEEDED",
"TOBACCO PRODUCT USER COUNSELLED",
"TOBACCO PRODUCTS USER (YES)",
"TOBACCO PT DECLINES DISCUSSION W/PROV",
"TOBACCO PT DESIRES DISCUSSION W/PROVIDER",
"TOBACCO QUIT LINE REFERRAL",
"TOBACCO REFERRAL",
"TOBACCO REFERRAL NOT OFFERED",
"TOBACCO REFERRAL OFFERED",
"TOBACCO REFERRAL REFUSED",
"TOBACCO REFUSED TO QUIT",
"TOBACCO SCREEN COMPLETED",
"TOBACCO SCREEN DECLINES",
"TOBACCO SCREEN FY09 BROCHURE",
"TOBACCO SCREEN POSITIVE",
"TOBACCO SET QUIT DATE",
"TOBACCO STOP SMOKING CLINIC OFFERED",
"TOBACCO STOP SMOKING CLINIC-PT AGREED",
"TOBACCO STOP SMOKING CLINIC-PT REFUSED",
"TOBACCO STOP SMOKING MEDS PT INTERESTED",
"TOBACCO STOP SMOKING MEDS PT REFUSED",
"TOBACCO SUPPORT SYSTEM REVIEWED",
"TOBACCO USE COUNSELED",

"TOBACCO USE COUNSELING",
"TOBACCO USE EDUCATION DECLINED",
"TOBACCO USE NEGATIVE PAST 30 DAYS",
"TOBACCO USE POS REFER TO CESSATION",
"TOBACCO USE POSITIVE 4 OR LESS CIGARETTE",
"TOBACCO USE POSITIVE >4 CIGARETTE",
"TOBACCO USE POSITIVE COUNSELING REFUSED",
"TOBACCO USE POSITIVE COUNSELING YES",
"TOBACCO USE POSITIVE DAILY CIGAR",
"TOBACCO USE POSITIVE NOT USING DAILY",
"TOBACCO USE POSITIVE SMOKELESS",
"TOBACCO USE PT CURRENT USER",
"TOBACCO USE/SMOKING SCREEN",
"TOBACCO USER",
"TOBACCO USER INPATIENT",
"TOBACCO USER OFFERED CLASSES",
"TOBACCO USER OFFERED MEDS",
"TOBACCO USER REFERRED TO PROVIDER",
"TOBACCO USER*",
"TOBACCO-ALREADY ON QUIT SMOKE MEDS/PROG",
"TOBACCO-PT READY TO QUIT",
"TOBACCO: ALREADY IN PROGRAM",
"TOBACCO: ALREADY ON MEDS",
"TOBACCO: DECLINES CLINIC REFERRAL",
"TOBACCO: DECLINES MEDICATIONS",
"Tobacco Counsel/Clinic/Meds Refused",
"Tobacco cessation referral refused",
"V 16 CURRENT TOBACCO USER",
"V 16 TOBACCO CESSATION > 12 MONTHS",
"V1-BARRIERS TO QUIT TOBACCO IDENTIFIED",
"V1-IDENTIFY SOC SUPPORT TO QUIT TOBACCO",
"V1-LUNG CA SCN HX >30 PACK YEARS",
"V1-PT ADVISED TO SET A QUIT TOBACCO DATE",
"V1-PT DECLINES REF TO TOBACCO CESS PRGM",
"V1-PT DECLINES TOBACCO CESSATION MEDS",
"V1-PT NOT INTERESTED IN QUIT TOBACCO USE",
"V1-PT READY TO QUIT TOBACCO USE",
"V1-PT RECEIVES TOBACCO CESS MEDS OUTSIDE",
"V1-PT REF TO NON-VA TOBACCO CESS PRGM",
"V1-PT THINKING ABOUT QUIT TOBACCO USE",
"V1-REASONS TO QUIT TOBACCO USE REVIEWED",
"V1-TOBACCO CESS MEDS NOT PRESCRIBED",
"V1-VARENICLINE MH DISORDER - CONTRA",
"V1-VARENICLINE MH DISORDER - RELAPSE",
"V1-VARENICLINE NO MH DISORDER - RELAPSE",
"V1-VARENICLINE NO UNSTABLE MH DX-RELAPSE",

"V1-VARENICLINE REFILL MH - RELAPSE",
"V1-VARENICLINE REFILL NO MH - CONTRA",
"V1-VARENICLINE REFILL NO MH - RELAPSE",
"V16 ATTENDING TOBACCO CESSATION PROGRAM",
"V16 CURRENT SMOKER",
"V16 CURRENT TOBACCO USER",
"V16 OFFERED TOB CESS MEDS",
"V16 TOB COUNSELING BY PROVIDER",
"V16 TOBACCO CESSATION < 12 MONTHS",
"V16 TOBACCO CESSATION <12 MONTHS",
"V16 TOBACCO CESSATION ED (PROVIDER)",
"V16 TOBACCO CESSATION PROGRAM DECLINED",
"V16 TOBACCO CESSATION PROGRAM REFERRAL",
"V16 TOBACCO CESSATION<12 MONTHS",
"V16 TOBACCO EDUCATION REFUSED",
"V16 TOBACCO MEDS ALREADY",
"V16 TOBACCO MEDS DECLINED",
"V16 TOBACCO MEDS OFFERED",
"V16 TOBACCO USE EDUCATION",
"V16 TOBACCO USE SCREEN",
"V16 UNABLE TO RESPOND TO TOBACCO SCRN",
"V3 TOBACCO QUIT <12 MOS COUNSELING",
"V7-NO TOBACCO USE > 7 YEARS",
"WILLING TO QUIT: DECLINES MEDS",
"WILLING TO QUIT: NO",
"WILLING TO QUIT: YES",
"YES-INTERESTED IN TOBACCO CESSATION",
"ZZIDENTIFY SOC SUPPORT TO QUIT TOBACCO",
"ZZV1-PT ASSISTED WITH TOBACCO CESSATION",
"ZZV1-PT READINESS TO QUIT TOB ASSESSED."

1.C. Atherosclerosis Cardiovascular Disease (ASCVD) events

The same approach as 1.B. was employed to define ASCVD events, MI, stroke, and Cardiac deaths using ICCD 9 and 10 diagnosis and procedure codes. When the VA women had unknown or no ICD codes for the event, we imputed the corresponding ASCVD event as no event.

Table S1. Akaike Information Criteria (AIC) and C-statistics of models with inclusion and exclusion of Ln age interaction terms stratified by race.

	**Non-Hispanic VA White women**			**Non-Hispanic VA African American (AA) women**		
**Models**	Without Ln age interaction terms †	ACC/AHA AA women model	The study model	Without Ln age interaction terms †	ACC/AHA white women model	The study model
**AIC***	43934.41	43931.90	43932.76	35959.82	35954.94	35953.19
**C-statistics**	0.62	0.62	0.64	0.63	0.63	0.63

ACC/AHA = American College of Cardiology/American Heart Association; AIC = Akaike Information Criteria

Notes. * Smaller AIC values are better. Models were estimated using Cox proportional hazard model.

† Without Ln age interaction terms model includes all CVD risk factors but excludes interaction terms with Ln age; The covariates include Ln age, Ln Systolic Blood Pressure (Ln SBP), ln SBP x on Antihypertensive medication, On Antihypertensive medication, Diabetes, current smoking, Ln total cholesterol, Ln High Density Lipoprotein (Ln HDL).

Table S2. Ten-year Atherosclerotic cardiovascular event risks in white, African American and Hispanic VA women.

	White	African American	Hispanics	
			White model	AA model
$S(10)^*$	0.9410	0.9391	0.9494	0.9494
10-year ASCVD risk (%)†	5.098%	5.157%	5.145%	5.207%

CVD = cardiovascular disease; SBP = Systolic Blood Pressure; HDL = High Density Lipoprotein; Ln = Natural log

Notes. * S(10) is 10-year CVD event free survival probability.

†. 1- S(10) ^ $e^{(x\beta-\bar{x}\beta)}$, where x a vector of covariates in the model and $\bar{x}$ mean values of corresponding covariates, and β is a vector of risk coefficients corresponding covariates, x . Specific values of x chosen to calculate 10-year CVD risk are age 50, total cholesterol 203 mg/dL, High Density Lipoprotein (HDL) 50 mg/dL, Systolic Blood Pressure (SBP) 120 mmHg, no diabetes, and no current smoking status.

Table S3. Estimates of Veterans Affairs (VA) Women, aged 40-79, Atherosclerotic Cardiovascular Disease (ASCVD) model by white and African American.

	White		*African American (AA)*	
	Est	SE	Est	SE
Ln Age	-25.063	17.613	-15.613	9.864
Ln Age, squared	2.170	1.303	---	
SBP untreated	0.823	0.212	-13.150	12.432
Ln SBP untreated x Ln Age	---		2.106	1.945
SBP treated	0.012	0.011	-0.660	0.652
Ln SBP treated X Ln Age	---		0.108	0.102
Diabetes	0.143	0.054	0.194	0.057
Current smoking	-3.514	2.670	-0.019	0.067
Current smoking X Ln Age	0.558	0.416	---	
Ln Total cholesterol	-0.867	5.795	0.235	0.134
Ln Total cholesterol X Ln Age	0.0158	0.902	---	
Ln HDL	2.827	4.144	-11.713	4.767
Ln HDL X Ln Age	-0.627	0.646	1.656	0.749
**C-statistics**	0.622		0.629	

Est = Estimate; HDL = High Density Lipoprotein; Ln = Natural log; SBP = systolic Blood Pressure; SE = Standard Error

Figure S1. HDL relative hazard of Atherosclerotic Cardiovascular Disease (ASCVD) risk by white and African American Veterans Affairs (VA) women.

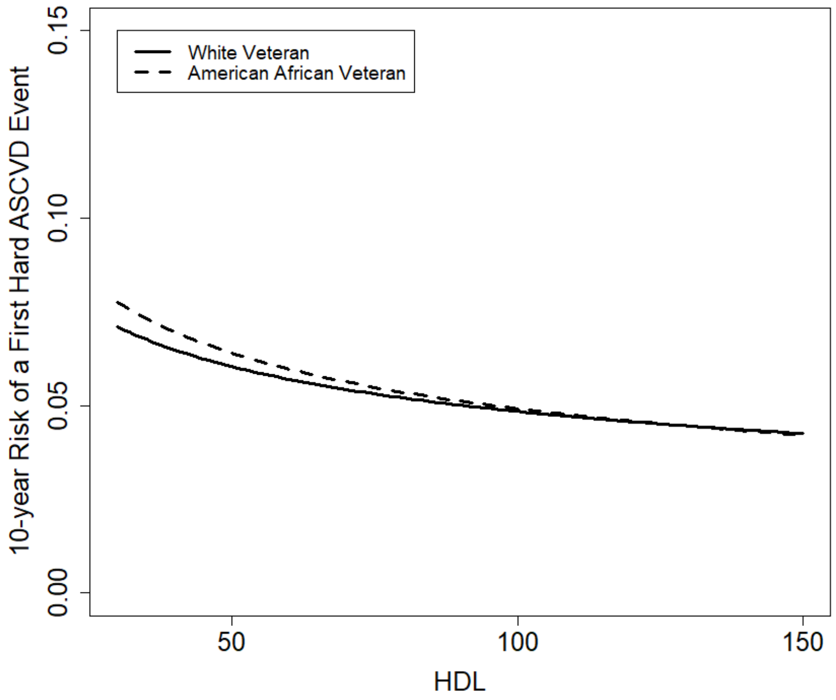

VA = Veterans Affairs; HDL = High Density Lipoprotein

*A solid line represents VA White women Atherosclerotic Cardiovascular Disease (ASCVD) risk assessment model; A dashed line represents the VA African American women model.

Figure S2. Hazard ratio of current cigarette smoking for Atherosclerosis Cardiovascular Disease (ASCVD) risk with aging in white Veterans Affairs (VA) women.

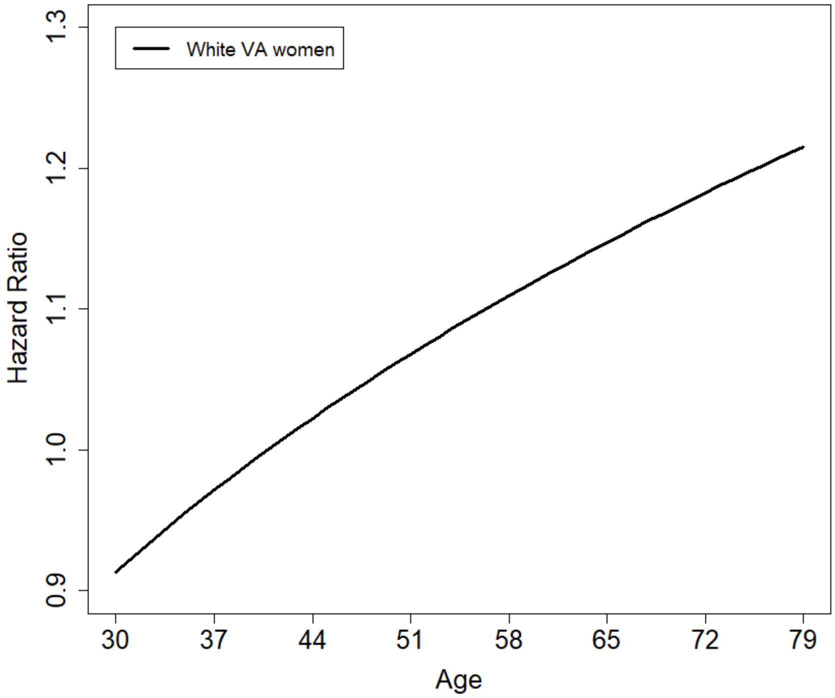

Figure S3. Aging effect on increased 10-year Atherosclerotic cardiovascular disease risk stratified by race between civilian women and women military service members aged 40-79 years old following ACC/AHA model structure.

3.A. White women

3.B. African American (AA) women

3.A. White women **3.B. African American (AA) women**

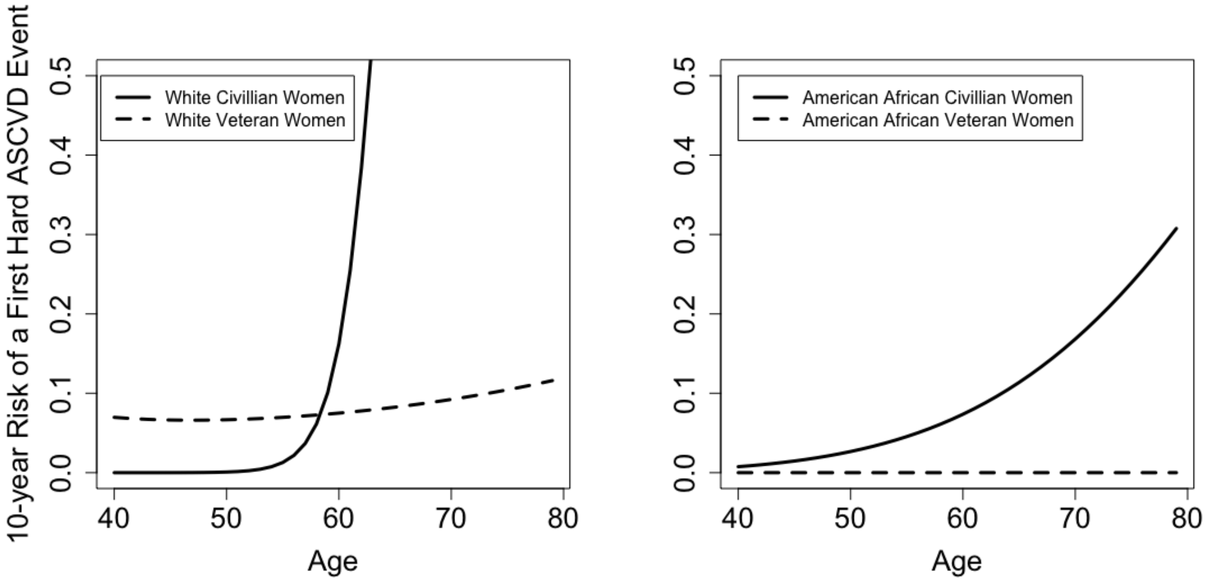

VA = Veterans Affairs; ASCVD = Atherosclerotic Cardiovascular Disease; ACC/AHA = American College of Cardiology/American Heart Association

Note. **Solid lines represent Civilian women Atherosclerosis Cardiovascular Disease (ASCVD) risk assessment model; Dashed lines represent the VA women model.
